# Improving the Diagnosis of Phenylketonuria by Using a Machine Learning–Based Screening Model of Neonatal MRM Data

**DOI:** 10.3389/fmolb.2020.00115

**Published:** 2020-07-07

**Authors:** Zhixing Zhu, Jianlei Gu, Georgi Z. Genchev, Xiaoshu Cai, Yangmin Wang, Jing Guo, Guoli Tian, Hui Lu

**Affiliations:** ^1^Center for Biomedical Informatics, Shanghai Children's Hospital, Shanghai Jiao Tong University, Shanghai, China; ^2^Shanghai Engineering Research Center for Big Data in Pediatric Precision Medicine, Shanghai, China; ^3^Department of Bioinformatics and Biostatistics, SJTU-Yale Joint Center for Biostatistics, Shanghai Jiao Tong University, Shanghai, China; ^4^Bulgarian Institute for Genomics and Precision Medicine, Sofia, Bulgaria; ^5^Newborn Screening Center, Shanghai Children's Hospital, Shanghai, China

**Keywords:** phenylketonuria, machine learning, newborn screening, MRM, logistic regression analysis (LRA)

## Abstract

Phenylketonuria (PKU) is a common genetic metabolic disorder that affects the infant's nerve development and manifests as abnormal behavior and developmental delay as the child grows. Currently, a triple–quadrupole mass spectrometer (TQ-MS) is a common high-accuracy clinical PKU screening method. However, there is high false-positive rate associated with this modality, and its reduction can provide a diagnostic and economic benefit to both pediatric patients and health providers. Machine learning methods have the advantage of utilizing high-dimensional and complex features, which can be obtained from the patient's metabolic patterns and interrogated for clinically relevant knowledge. In this study, using TQ-MS screening data of more than 600,000 patients collected at the Newborn Screening Center of Shanghai Children's Hospital, we derived a dataset containing 256 PKU-suspected cases. We then developed a machine learning logistic regression analysis model with the aim to minimize false-positive rates in the results of the initial PKU test. The model attained a 95–100% sensitivity, the specificity was improved 53.14%, and positive predictive value increased from 19.14 to 32.16%. Our study shows that machine learning models may be used as a pediatric diagnosis aid tool to reduce the number of suspected cases and to help eliminate patient recall. Our study can serve as a future reference for the selection and evaluation of computational screening methods.

## Introduction

Phenylketonuria (OMIM:261600) (PKU) is a common inborn genetic metabolic disorder, which affects the infant's neural development and manifests as abnormal behavior and developmental delay, which becomes apparent as the child grows. In China, the incidence of PKU has a wide range [between 1/3,420 Wang et al., 2015 and 1/26,668 Fan et al., 2009], and the prevalence estimates are still increasing (Gu and Wang, 2004). There are several medical tests that are used for PKU neonatal screening such as the Guthrie test (Guthrie and Susi, 1963), and tests utilizing high-performance liquid chromatography (Moretti et al., 1990) and tandem mass spectrometry (MS/MS) (Rashed et al., 1995). In most countries around the world, PKU diagnosis is performed by evaluating phenylalanine (Phe) and tyrosine (Tyr) levels in neonatal dry blood spots (DBSs) by MS/MS (Blau et al., 2014). Following a positive initial test result, the presence of Phe must be confirmed with immediacy by a repeat screening.

A triple–quadrupole mass spectrometer [multiple reaction monitoring (MRM)] is used to measure 44 metabolites in neonatal blood samples, and in clinical practice, more than 30 types of genetic metabolic diseases (including PKU) are diagnosed by using these biomarkers. However, the initial PKU screening by MRM is characterized by a high false-positive rate. Thus, clinicians have to recall a large number of false-positive patients for rescreening and further testing, which increases the pressure of medical resources and creates an additional economic and time burden on patients. Furthermore, an estimated 13% positive predictive value (PPV) in PKU neonatal screening (Zhang et al., 2019) indicates that the pathophysiology of the disease is not uniquely driven by elevated level of Phe and Tyr. Additionally, the large number of metabolites captured in experimental MRM data creates inherent complexity, which makes the overall meaningful signals non-trivial to assess by a manual process for a sizable patient population.

Machine learning methods have the advantage of utilizing high-dimensional and complex features, which can be obtained from the patient's metabolic patterns and interrogated to reduce the PKU diagnostic test's false-positive rate. New factors can also be discovered to aid PKU diagnosis without *a priori* knowledge. In recent years, several machine learning techniques have been used to map metabolomics databases (Baumgartner et al., 2004a), and machine learning methods have been used to construct classification models with high diagnostic prediction (Baumgartner et al., 2004b, 2005; Chen et al., 2013) [further review Cuperlovic-Culf, 2018]. Most of those models are utilized to predict normal vs. disease states and use metabolic patterns in newborn screening MRM data to develop the classifier. At the same time, such models may be well-positioned to discover potential disease biomarkers from MRM-based high-dimensional metabolic data (Mendes, 2002). For example, Baumgartner and colleagues developed several classification models (Mendes, 2002) and identified metabolites with abnormally changed concentrations (Baumgartner et al., 2005). Moreover, some of the models have been able to predict cases within the suspected group, which were diagnosed initially as positive (exceeding screening cutoff values in initial screen by a widely used cutoffs scheme) but finally diagnosed as negative cases, and cases that had values over the screening cutoffs and diagnosed subsequently as positive cases (Chen et al., 2013).

However, such computational models are yet to be widely applied in pediatric clinical practice. Initial clinical screening for PKU is well-established and mature process; thus, the more pressing problem to be solved for this rare but treatable metabolic disorder is to develop and fine-tune classification models that pinpoint false-positives and reduce the number of suspected cases to be subject to subsequent screening and verification while ensuring that no false-negatives occur.

To address the above stated unmet need in PKU clinical screening, we employed feature selection strategies and utilized logistic regression analysis (LRA) techniques, together with metabolic data of more than 600,000 newborn screenings, to develop machine learning–based screening models for PKU. Our goal is to minimize false-positive cases, maximize specificity (*S*_*p*_), and to serve as a guiding reference for the selection and evaluation of future clinically relevant screening methods.

## Materials and Methods

### Patient Metabolic Data

Dried blood samples are routinely collected from newborn babies at the Newborn Screening Center of Shanghai Children's Hospital for the purpose of PKU screening. Three to four DBSs are blotted from the infant's heel, and each blood spot is ~1 cm in diameter. Subsequently, a tandem mass spectrometry system (MRM) is used, including mass spectrometer (MSMS, Waters Quattro micro, Milford, Massachusetts, USA), a high-performance liquid analyzer (Waters 1525 u), an automatic sampling system (Waters 2777 Sample manager), and a non-derivative tandem mass spectrometry kit (NeoBase™ Non-derivatized MSMS Kit; PerkinElmer, Waltham, Massachusetts, USA) to measure 11 amino acids, 32 acylcarnitines, and succinylacetone (Table 1), and the values are entered in the hospital's computer system. For this historical retrospective study, we obtained records for 633,997 newborns, which were collected from 2010 to 2018. Each record contained measurements for the level of the 44 metabolites and a clinician-entered binary field indicating the patient's overall PKU diagnosis. The data were sourced from the hospital data warehouse, and to protect the privacy and anonymity of the patients, all patient-identifying information was removed and obfuscated prior proceeding with the analytical treatment.

**Table 1:** Metabolites detected by MRM analysis in newborn screening.

**Amino acids (11)**		
Alanine (Ala)	Glycine (Gly)	Phenylalanine (Phe)
Arginine (Arg)	Methionine (Met)	Proline (Pro)
Citrulline (Cit)	Ornitine (Orn)	Tyrosine (Tyr)
Valine (Val)	Leucin/isoleucine/hyrdoxyproline (Leu/IIe/Pro-OH)	
**Fatty acids (31)**		
Free-carnitine (C0)		Dodecanoyl-carnitine (C12)
Acetyl-carnitine (C2)		Dodecenoyl-carnitine (C12:1)
Propionyl-carnitine (C3)		Myristoyl-carnitine (C14)
Malonyl-carnitine+3-Hydroxybutyryl-carnitine (C3DC_C4OH)		3-Hydroxytetradecadienoyl-carnitine (C14-OH)
Butyryl-carnitine (C4)		Myristoleyl-carnitine (C14:1)
Methylmalonyl-carnitine+3-Hydroxyisovaleryl-carnitine (C4DC_C5OH)		Tetradecadienoyl-carnitine (C14:2)
Isovaleryl-carnitine (C5)		Hexadecanoyl-carnitine (C16)
Tiglyl-carnitine (C5:1)		3-Hydroxypalmitoyl-carnitine (C16-OH)
Glutaryl-carnitine+3-Hydroxyhexanoyl-carnitine (C5DC_C6OH)		Hexadecenoyl-carnitine (C16:1)
Hexanoyl-carnitine (C6)		3-Hydroxypalmitoleyl-carnitine (C16:1-OH)
Methylglutaryl-carnitine (C6-DC)		Octadecanoyl-carnitine (C18)
Octanyl-carnitine (C8)		3-Hydroxystearoyl-carnitine (C18-OH)
Octenoyl-l-carnitine (C8:1)		Octadecenoyl-carnitine (C18:1)
Decanoyl-carnitine (C10)		3-Hydroxyoleyl-carnitine (C18:1-OH)
Decenoyl-carnitine (C10:1)		Octadecadienoyl-carnitine (C18:2)
Decenoyl-carnitine (C10:2)		
**Ketones (1)**	
Succinylacetone (SA)		

**Figure 1:**
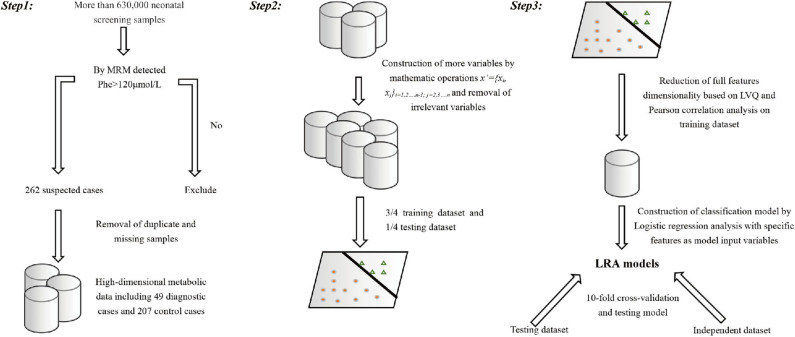
Visual depiction of the analytical workflow from data collection, through features selection to model development and evaluation.

Full dataset comprised 633,997 patient cases, including 326,508 boys and 307,489 girls. The average age at the time of blood collection was 3.6 days (range, 2–30 days), and the average weight was 3.3 kg (range, 1.73–4.89 kg) (Table 2). We applied a popular PKU screening cutoff level of Phe >120 μmol/L, and patients fulfilling this criterion were included in the reduced dataset of 262 records. Six records were removed Because of data duplication for a final dataset of 256 records of PKU-suspected cases. This screening cutoff is also applied in the course of clinical practice at the Shanghai Children's Hospital; thus, these 256 newborns were also recalled for additional DBS testing at the time that the PKU suspicion was established. Newborns with DBS screening value again above the screening cutoff were then requested to participate in a confirmation test (including urine tests, blood tests, genetic tests). Of the 256 suspected cases, 49 were finally diagnosed with PKU. Thus, our dataset utilized in the model development in this study consisted of 49 positive-labeled examples (PKU-suspicion confirmed) and 207 negative-labeled examples (PKU-suspicion rejected). Figure 1 visualizes the model development process in this work.

**Table 2:** The characteristics of newborn babies.

	**Total**	**Suspected**	**Control**	**PKU**
No. of samples	633,997	256	207	49
**Sex**				
M	326,508	126	104	22
F	307,489	130	103	27
Average age at blood collection	~3.6 days (2–30 days)			
Birth weight	3.3	3.3	3.3	3.2
	(1.73–4.89)	(1.75–4.87)	(1.77–4.87)	(1.75–4.7)
Gestational age	~39.13 week (30–44 week)			

### Features and Feature Selection Strategy

Starting with the variables representing the level of the metabolites measured by MRM, we constructed additional variables by mathematical operation (step 1). All samples are randomly divided into a training set (3/4) and a test set (1/4). A combination of analytical methods was applied on the training set (steps 2–4) to reduce the feature set with the goal to exclude highly correlated and irrelevant features in order to improve the model performance, prevent overfitting, and select the optimal feature combination for building an optimized model.

Step 1:

Additional feature variables *x*′ were constructed such that:

$$\begin{array}{l} x^{\prime }=\;\left[x_{i}/x_{j}\right];i=1,2,\ldots .\left(n-1\right);j=2,3\ldots .n \end{array}$$

where *x*′ represents the new feature variable, *x*_*i*_*, x*_*j*_ is the variable representing metabolite measured by MRM, and “/” represents the ratio of the two variables.

We considered this as a suitable strategy, because the Phe/Tyr ratio is a widely used clinical indicator, and metabolite level ratios have been used in previous studies (Chen et al., 2013). The expanded feature set contained more than 700 candidate features.

Step 2:

Learned vector quantization (LVQ) (Kohonen, 1998, 2001) is a self-organizing neural network model, based on supervised learning, which consists of a competition layer and a linear layer. The LVQ algorithm, as implemented in the *caret* (Max, 2008) R package, was applied to rank the features importance (calculated by the varImp() function). The top two ranked features with the highest receiver operating characteristic (ROC) curve variable importance were selected. In addition, two diagnostic standard features for clinical biomarker diagnosis of PKU were added. These features were (Met/Phe, Phe/Tyr, Phe, Tyr).

Step 3:

A linear relationship between the variables is measured by using the Pearson correlation coefficient (Pearson, 1895). Pearson correlation analysis was used to further adjust feature selection and remove highly correlated features.

Step 4:

We used the Wilcoxon rank sum test to evaluate whether the metabolite concentration represented by the selected features was significantly different in the positive and negative labeled sets.

Step 5:

Logistic regression analysis is widely used in biomedical applications (Pearson, 1895). To increase our model's clinical interpretability, we constructed a classification model on diagnostic flags using LRA.

### Model Training and Evaluation of Model Performance

#### Model Training

We constructed four LRA models (LRA1–LRA4) from different feature set combinations (Table 3) and calculated the Addictive Net Reclassification Index (Add NRI) and Absolute Net Reclassification Index (Abs NRI) (Hosmer and Lemeshow, 2000) for the comparison of each model. The models were computed utilizing the R *glm* function.

**Table 3:** The four models developed and the corresponding combination of selected features.

**Model**	**Feature combination**
LRA1	Phe
LRA2	Phe, Tyr
LRA3	Phe, Tyr, Met/Phe
LRA4	Met/Phe

#### Comparison With Previous Work

To compare our results with existing results in the literature, we calculated an additional fifth model (LRA5) (Table 4), which utilized the optimal feature set developed in a 2013 study by Chen et al. (2013).

**Table 4:** Model developed utilizing features from previous work (LRA5) and our optimal model (LRA3).

**Model**	**Feature**
LRA3	Phe, Tyr, Met/Phe
LRA5	Met, Phe, C4, Ala, Eu × Tyr, C16:1

#### Cross-Validation on Testing Set and Validation on an Independent Dataset

We used a 10-fold cross-validation method to evaluate the stability of the classification model and determine that the model has achieved sufficient statistical performance on the testing dataset. We used *S*_*n*_, *S*_*p*_, PPV (precision), negative predictive value (NPV), accuracy (Acc), and area under curve (AUC) as measurements to evaluate the discriminatory power of the classification models. These metrics were calculated as follows: *S*_*n*_ = TP/(TP + FN); *S*_*p*_ = TN/(TN + FP); PPV = TP/(TP + FP); NPV = TN/(TN + FN). *S*_*n*_ expresses how the proportion of true-positive cases detected, *S*_*p*_ indicates the proportion of negative results in test cases without the disease. Additionally, there were 111 suspected cases with Phe >120 that were used to validate the model, including 37 PKU patients and 74 false-positive patients. The male-to-female ratio was ~1:1.13; the average age at the time of blood collection was 4.5 days, and the average weight was 3.4 kg.

## Results

### Metabolic Dataset Exploration and Visualization

**Figure 2:**
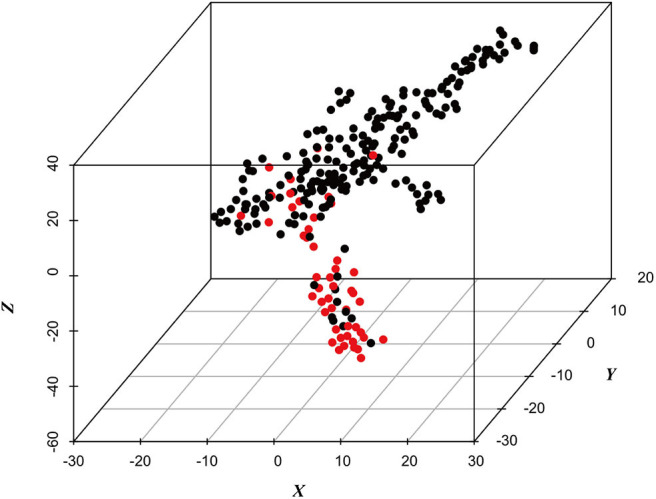
Visualization of the metabolic data set computed by t-SNE. Red signifies classification as positive, and black signifies classification as negative.

Using the machine learning visualization methods t-SNE (Li et al., 2017), we calculated a visualization of the structure of the dataset. The three-dimensional figure computed by t-SNE (Figure 2) illustrates that there are false-positives interspersed within the negative class space, and those can be excluded using machine learning–based analysis.

### Feature Selection, Model Development, and Evaluation

#### Feature Selection

**Figure 3:**
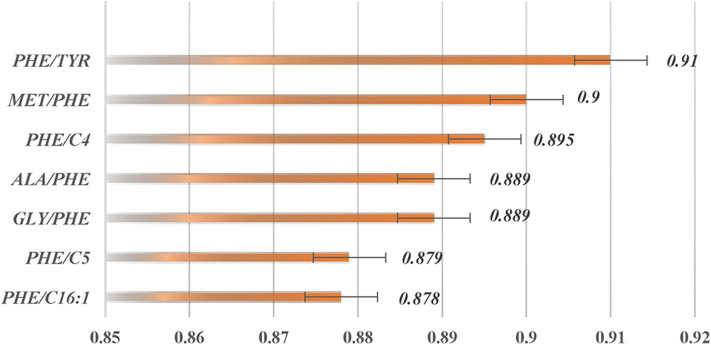
Ranking of feature importance calculated by *caret* package in R utilizing LVQ analysis.

The two top-ranked features by LVQ were Met/Phe, Phe/Tyr (Figure 3), and Phe, and Tyr as clinical biomarkers was considered. In addition, correlation analysis with cutoff >0.8 was used to remove highly correlated features, and as a result, Phe/Tyr was excluded. By applying a Wilcoxon test, we evaluated the means of the positive and negative groups for each corresponding feature for statistically significant difference. Table 5 summarized the results of the means computations and test results.

**Table 5:** Average values and standard deviations for the selected feature variables and results of the Wilcoxon rank sum test.

**Features**	**Mean** **±** **SD**		**Wilcoxon rank sum test**	***p***
	**Control (μmol/L)**	**PKU (μmol/L)**		
	**(*n* = 207)**	***n* = 49**		
Met/Phe	0.29 ± 0.45	0.055 ± 0.054	9,283	<2.2e-16
Phe	216.01 ± 231.96	898.58 ± 696.91	1,127	<2.2e-16
Tyr	164.18 ± 179.43	66.87 ± 26.16	8,150	4.001e-11

#### Model Performance

**Figure 4:**
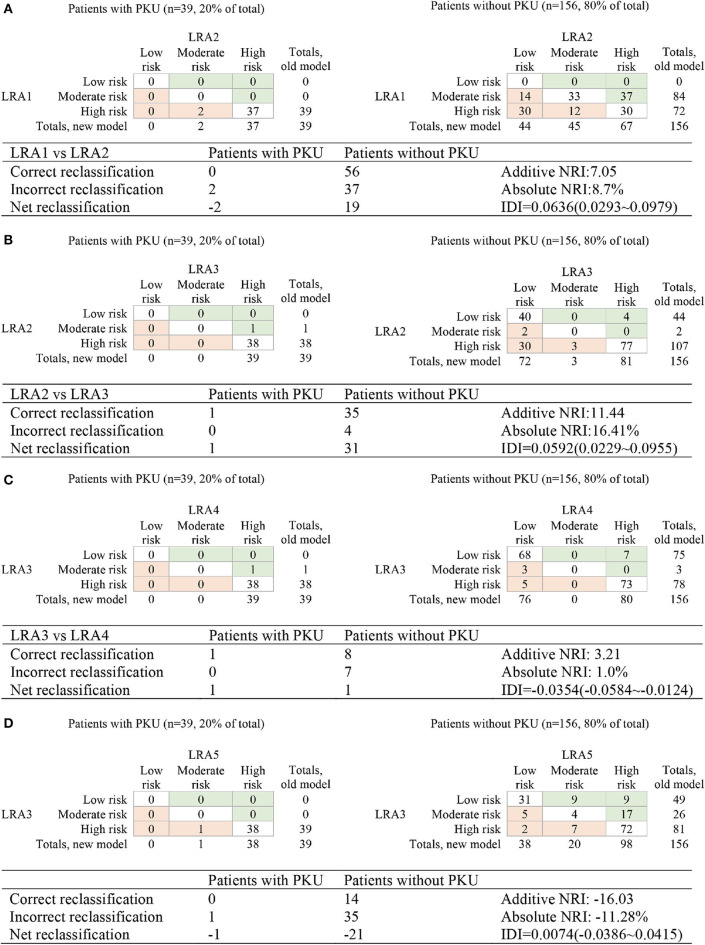
Reclassification of risk for the comparison of the performance of LRA1–LRA5. **(A)** Compare LRA1 and LRA2; the low risk <0.0528, medium risk 0.528–0.0948, and high-risk >0.0948. **(B)** Compare LRA2 and LRA3; the low risk <0.0528, intermediate risk 0.0528–0.0579, and high risk >0.0579. **(C)** Compare LRA3 and LAR4; the low risk <0.0220, medium risk 0.0220–0.0579, and high risk> 0.0579. **(D)** Compare LRA3 and LAR5; the low risk <0.0220, medium risk 0.0220–0.0579, and high risk >0.0579.

Classification models (LRA1–LRA5) (Table 6) were trained on the training dataset containing *n* positively labeled cases of disorder (PKU-positive: *n* = 39) and *m* negatively labeled cases (PKU-negative, *m* = 156). The 156 cases were originally clinically suspect for PKU but were diagnosed as PKU-negative in additional clinical screening. Figure 4 summarizes the comparison results of the performance of each model.

**Table 6:** LRA1–LRA5 classification models.

**Model**	**Logit of model *z* = β_0_+β_1_x_1_+β_2_x_2_+…+β_i_x_i_**	**OR (95% CI)**	***Z***	***p***
LRA1 (Phe)	−2.6068 + 0.0029·Phe	1.0032 (1.0021–1.0046)	5.517	3.45e-08^*^
LRA 2 (Phe, Tyr)	−0.5046 + 0.0025·Phe – 0.0207·Tyr	Phe = 1.0025 (1.0016–1.0037), Tyr = 0.9751 (0.9593–0.9881)	4.269 −3.042	1.96e-05^*^ 0.0024^*^
LRA 3 (Met/Phe, Phe, Tyr)	0.7722 – 13.2300·Met/Phe + 0.0010·Phe – 0.0090·Tyr	Met/Phe = 1.79e-06 (4.19e-11–0.009)	−2.720	0.0065^*^
LRA4 (Met/Phe)	1.2661 – 21.4822·Met/Phe	3.76e-10 (8.33e-14–3.58e-07)	−5.485	4.13e-08^*^
LRA5 (Met, Phe, C4, Ala, Eu × Tyr, C16:1)	0.7997 + 1.282e-03·Ala – 7.329e-02·Met + 2.877e-03·Phe – 4.531·C4 −6.102·C16:1 −7.559e-06·Eu × Tyr	Ala = 1.0010 (9.97e-01–1.0037), Met = 0.9286 (0.8750–0.9797), Phe = 1.0028 (1.0017–1.0040), C4 = 0.0069 (1.38e-05–0.5384), C16:1 = 0.0097 (1.96e-07–42.85), Eu × Tyr = 1.0000 (0.9999–1.00001)	0.602 −2.393 4.424 −1.819 −0.953 −0.266	0.5474 0.0167^*^ 9.7e-06^*^ 0.0689 0.3405 0.7899

* *p < 0.05 were selected*.

We calculated and compared reclassification of risk between PKU patients and false-positive patients in the LRA1–LRA5 models to determine the performance of different models in screening PKU false-positive samples. The optimal model LRA3 with the optimal feature set was characterized with the results of risk reclassification (Figures 4B–D). The features included in this model were traditional biomarkers Phe, and Tyr, and the new potential biomarker Met/Phe. More PKU patients are all subject to a higher risk assessment, and more non-PKU patients were reclassified to a lower risk assessment.

In this analysis, both LRA1 and LRA2 models (Table 3) were constructed using the traditional clinical screening markers of PKU. The feature(s) included in LRA1 was Phe, and in LRA2, Phe and Tyr. We started by comparing LRA1 and LRA2. According to the classification threshold of the resulting event, we set the low risk to <0.0270, the medium risk to between 0.0270 and 0.101, the high risk to >0.101 and then calculated Add NRI and Abs NRI (Figure 4A). Model LRA2 performed better than LRA1. Choosing the better performing model from the above results for comparison with LRA3 (Figure 4B), we also set thresholds according to the resulting events (low risk <0.0270, medium risk 0.0270–0.0307, and high risk > 0.0307). The results of risk reclassification showed that one PKU patient received a higher risk assessment, and another 26 false-positive patients received a low risk assessment.

We compared LRA3 with LRA4 (which included the features Met/Phe) (Figure 4C) and LRA5 [which was selected from literature (Chen et al., 2013) and included the features Met, Phe, C4, Ala, Eu × Tyr, and C16:1] (Figure 4D). The cutoff values of the resulting event used in the LRA4 comparison were low risk <0.0307, medium risk 0.0307–0.0336, and high risk >0.0336, and in the comparison with LRA5: low risk <0.0067, medium risk 0.0067–0.0307, and high risk >0.0307. The results of the risk reclassification show that there is no obvious difference between the performance of LRA3 and LRA4, but compared with LRA5, all 39 PKU patient samples are subject to a higher risk assessment in the LRA3 model (Figures 4C,D).

#### Cross-Validation and Independent Validation

**Figure 5:**
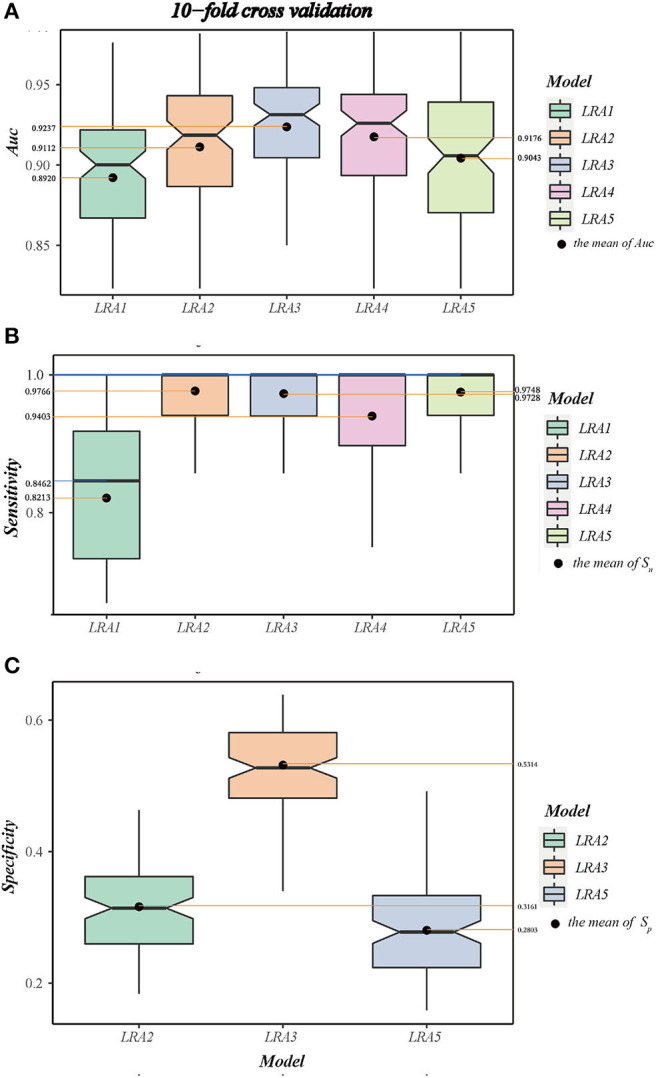
**(A)** The boxplot A shows the area under curve (AUC) and value interval and relative stability of the LRA1–LRA5 models after 10-fold cross-validation. **(B)** The boxplot B shows the median and mean sensitivity, value interval, and relative stability of the LRA1–LRA5 models after 10-fold cross-validation. **(C)** The boxplot C shows the specificity and value interval and relative stability of the LRA2, LRA3, LRA5 models after 10-fold cross-validation.

A 10-fold cross-validation was used to examine and evaluate the classification performance of the LRA1–LRA5 models developed in this study (Figure 5 and Table 7). Except for the mean *S*_*n*_ of LRA1 and LRA4 models <95%, the other LRA models ensure that *S*_*n*_ is >95% (Figure 5B); the mean *S*_*p*_ improved compared to traditional screening methods to values ranging from 28.03% (LRA5) to 53.14% (LRA3). Positive predictive value increased from 19.14% to values ranging between 23.67% (LRA5) and 32.16% (LRA3).

**Table 7:** Classification performance of the LRA2–LRA4 classifiers.

**Model**	**Mean**					
	**S**_**n**_ **(%)**	**S**_**p**_ **(%)**	**PPV (%)**	**NPV (%)**	**Acc (%)**	**AUC (%)**
LRA1 (Phe)	82.13	69.48	40.42	94.95	71.41	89.20
LRA2 (Phe, Tyr)	97.66	31.61	24.59	98.49	43.77	91.12
LRA3 (Met/Phe, Phe, Tyr)	97.28	53.14	32.16	98.93	61.27	92.37
LRA4 (Met/Phe)	94.04	56.52	32.98	97.77	63.43	91.75
LRA5 (Met, Phe, C4, Ala, Leu × Tyr, C16:1)	97.48	28.03	23.67	98.35	40.82	90.43

Between-model comparison showed that all five models are feasible; however, LRA3 with feature set (Met/Phe, Phe, Tyr, and median and mean of AUC = 0.9313 and 0.9237) (Figure 5A) exhibited improved performance compared to the others as assessed by AUC. In addition, from a standpoint of improved screening performance, LRA3 showed improvement compared to the other models. Its *S*_*p*_ had the highest mean *S*_*p*_in the model with *S*_*n*_ > 95% (Table 7).

Next, in order to verify the reliability and applicability of our LRA models, independent data of control and diagnostic cases that satisfy the level of Phe >120 μmol/L were used to validate the model. All models expressed 100% *S*_*n*_, and the LRA3 model still had the highest screening performance with *S*_*p*_ of 39.19%.

## Discussion

The motivation for our analysis was to develop a suitable logistic regression-based machine learning model using metabolomics data, tuned to minimize the number of false-positives in PKU diagnosis during the PKU screening process. Our additional goal was to drive biomarkers discovery and thus to provide and improve precision medicine approaches in rare genetic diseases such as PKU and serve as a reference point for future implementations.

In this work, we sourced pediatric patients' metabolic data from MRM screening, applied the LVQ method to perform feature importance ordering, and used correlation analysis and logistic regression to establish an optimal classification algorithm. Overall, the results show that despite inherently noisy clinical data, meaningful features can be extracted from metabolic data to screen for false-positives in PKU. Significantly, reducing false-positives can lighten the workload of the screening medical professionals, improve detection efficiency, and reduce the cost and inspection time expenditure of pediatric patients and their caregivers. Supplementary screening models based on MRM data can also provide more efficient cohort identification for prospective studies.

### Data, Study Design, and Populations

Our data approach is distinctive from previous studies. In our design, the control group consists of high-risk individuals, which greatly reduces the unbalance problem of the dataset and reduces the possibility of overfitting of the model. In contrast, other works employing development of decision trees (DT), support vector machines (SVM), artificial neural networks (ANN), k-nearest neighbor classifier (k-NN), discriminant analysis (DA), and LRA models use normal and disorder patient cases in order to perform such classification in neonatal screening (Baumgartner et al., 2004a,b, 2005).

Our work utilizes a dataset that is Chinese-focused, an approach motivated by the potential differences between Chinese and other ethnic groups. For example, the mutation spectrum of PKU in Chinese population is similar to other Asian populations but significantly different from European populations (Song et al., 2005). Furthermore, the prevalence of PKU varies geographically and ethnically from race to race; PKU birth prevalence per 10,000 live births was estimated to be 1.14 (0.96–1.33) among white, 0.11 (0.02–0.37) among black, and 0.29 (0.10–0.63) among Asian ethnic groups (Hardelid et al., 2008). The prevalence of PKU ranged from 0.005 to 0.0167% in Arab countries (El-Metwally et al., 2018). In China, the prevalence was estimated as 1 in 3,795 (Shi et al., 2012). Our work helps bridge this knowledge gap and provide insights that are applicable to the context of the Chinese health system.

### Feature Selection Strategy

Feature selection usually plays a key role in machine learning to exclude attributes, which may cause overfitting results in classification analysis and reduce interpretability (Bagherzadeh-Khiabani et al., 2016). The genetic metabolic disease data based on mass spectrometry have characteristics such as limited number of samples, many features, and noise interference, to name a few. Because of unrelated and redundant attributes, the traditional unsupervised dimensionality reduction methods do not use the label information effectively, so the subspaces they find may not be the most separable in the data (Liu et al., 2017). On the one hand, the feature subset selection can identify and remove as many irrelevant and redundant variables as possible, thereby reducing the data dimensions. By selecting only the relevant attributes of the data, the machine learning prediction accuracy and classification performance can be improved (Saeys et al., 2007; Walter and Tiemeier, 2009). On the other hand, it is also valuable for pediatric clinicians to know disease-influencing variables; those new variables can be validated and can become part of an updated screening plan. The selection of variables by experts and literature review, however, may introduce bias (Matalon and Michals, 1991).

In our models, we proposed to better understand which signals might be closely related to PKU. We applied a feature selection strategy that calculates the Euclidean distance between input sample and weight vector until it finds the prototype vector closest to the sample. All the variables were ranked according to the ROC curve variable importance in the LVQ algorithm, most of the top two features are indicated or used as screening markers for PKU, supporting the validity of this strategy. When new features are included in the model, NRI can be used to compare the performance between the original model and the model after incorporating the new features. For example, we compared LRA1 (Phe) and LRA2 (Phe, Tyr); here, LRA1 is the old model, and LRA2 is the new model (Figure 4). The new proposed features differ from traditional clinical indictors; thus, clinical validation will be necessary to establish the accuracy of these features. Some related evidence has already been reported (Chen et al., 2013).

### Model Differences

So far, several screening and classification models using machine learning methods have been reported for PKU (Baumgartner et al., 2004a,b, 2005; Chen et al., 2013). When applied to our dataset, these models performed with difference in results and achieved a low *S*_*p*_ ranging from 0.2752 to 0.4510. Our work chooses the LRA method, which is a traditional clinical model with high clinical interpretability. Our LRA model shows improved performance compared to existing models, with a cross-validation *S*_*p*_ = 0.5314 ± 0.0800. Another difference is that other studies have focused on constructing primary screening models. Such models perform less effectively if applied to our dataset.

### Odds Ratio Comparison

Odd ratios (ORs) is a commonly used indicator in case-control studies in epidemiology, reflecting the strength of the association between disease and exposure. A value of OR >1 indicates that the factor is a risk factor; if OR <1, the exposure to the factor is protective, and if the OR = 1, this indicates that the factor does not contribute to the occurrence of the disease. Table 5 shows the features OR of our model. An increase in the concentration of Phe may cause disease, which corresponds well to OR >1, whereas Tyr shows an OR <1 with decreasing levels. The new marker Met/Phe may be a factor rather similar as Tyr, which can be confirmed by clinical verification.

### Future Direction

The application of machine learning in metabolic diseases research continues to evolve and improve. Additional pediatric clinical data, such as the child's height, weight, gestational age, and aspects of family history and *-omics* data such as genomics, transcriptomics, and proteomics data can be incorporated in model development, which together with the analysis of correlation between multigroup data and clinical outcomes, is our goal in future work. Additionally, our data are sourced from a single center in Shanghai. In the future, a shared platform incorporating data from multiple sources and centers would be beneficial for medically relevant discovery based on a heterogeneous population. A basis for such platform would be the development of a standardized terminology system and a harmonization of instrumentation and diagnostic measures such as cutoff values in various clinical sites.

Machine learning methods are still in an emerging stage in the research and application in rare genetic metabolic diseases, and there are still many unsolved problems to be explored in the future. For example, whether it is equally possible to use machine learning applications in other rare genetic diseases or to discover new biomarkers or even new unknown metabolic pathways and biochemical reactions remains to be explored by future research.

## Summary

In this study, we applied multiple LRA models, a supervised machine learning algorithm for constructing method applicable in pediatric diagnostic screening in PKU utilizing high-dimensional metabolic data. These models achieved performance of guaranteed *S*_*n*_ >95%, achieved AUC higher than 90%, and improved *S*_*p*_ and PPV on both the test set and independent test set. We reported a new marker of PKU—Met/Phe. The model with a feature set combining this new marker with traditional biomarkers (Phe, Tyr) can reduce more than half of the false-positives. Our study can serve as a relevant reference for the selection and evaluation of PKU screening methods in pediatric medical practice.

## Data Availability Statement

The datasets generated for this study are available on request to the corresponding author.

## Ethics Statement

This study was approved by the Ethics Review Committee of the Shanghai Children's Hospital, Shanghai Jiao Tong University (Approval No. 2019R075-E01). Informed consent was obtained from a parent or guardian of each patient before newborn screening and the study.

## Author Contributions

ZZ initiated this research project. ZZ, YW, JGuo, and GT processed the data. ZZ and JGu designed this study. ZZ, GG, XC, GT, and HL conducted the statistical modeling and performed the data analysis. ZZ, GG, HL, and GT designed, wrote, and reviewed the manuscript. All authors read and approved the final manuscript.

## Conflict of Interest

The authors declare that the research was conducted in the absence of any commercial or financial relationships that could be construed as a potential conflict of interest.
